# Carbohydrate response element‐binding protein (ChREBP) mediates decreased SNAP25 expression in islets from diabetic Goto‐Kakizaki (GK) rats

**DOI:** 10.1002/2211-5463.13900

**Published:** 2024-09-19

**Authors:** Anyi Hu, Hongyan Lan, Zilai Yao, Xiangchen Kong

**Affiliations:** ^1^ Shenzhen University Diabetes Institute, School of Medicine Shenzhen University China

**Keywords:** ChREBP, insulin secretion, SNAP25, type 2 diabetes

## Abstract

SNAP25 plays an essential role in the glucose‐stimulated insulin secretion (GSIS) of pancreatic β‐cells. Carbohydrate response element‐binding protein (ChREBP) is an important transcription factor in β‐cells and, in this study, we aimed to explore whether ChREBP regulates SNAP25 expression in β‐cells. We show that diabetic Goto‐Kakizaki (GK) rats exhibited impaired insulin secretion and hyperglycemia, along with decreased SNAP25 expression and ChREBP phosphorylation in islets. SNAP25 knockdown decreased GSIS in β‐cells, while SNAP25 overexpression increased GSIS in β‐cells. Activation or overexpression of ChREBP led to reduced SNAP25 expression and subsequent suppression of GSIS. Conversely, ChREBP knockdown mitigated the reduction in SNAP25 expression caused by high glucose. Mechanistically, the activation of ChREBP by high glucose increased its occupancy and decreased the level of H3K4me3 at the *Snap25* promoter. Our findings reveal the novel regulatory mechanisms of SNAP25 expression in β‐cells and suggest that SNAP25 may be involved in the regulation of β‐cell secretory function controlled by ChREBP.

## Graphical abstract

SNAP25 plays an essential role in the glucose‐stimulated insulin secretion of pancreatic β‐cells. Diabetic hyperglycemia suppresses SNAP25 expression in β‐cells, an effect associated with the activation of ChREBP, leading to increased ChREBP recruitment to the promoter of *Snap25*, and subsequently reducing the tri‐methylation of histone H3 at lysine 4 (H3K4me3) at the promoter.

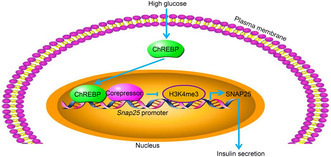

**Abbreviations**
ChoRE: carbohydrate response element
ChREBP: carbohydrate response element‐binding protein
GK: Goto‐Kakizaki
H3K4me3: tri‐methylation of histone H3 at lysine 4
SNAP25: synaptosomal‐associated protein 25
SNAREs: soluble *N*‐ethylmaleimide‐sensitive factor attachment protein receptors
T2D: type 2 diabetes

Blood glucose homeostasis is maintained by insulin secreted by pancreatic β‐cells. Insulin secretion is a highly regulated process that involves the exocytosis of insulin‐containing granules [1]. This process is orchestrated by a group of proteins known as SNAREs (Soluble *N*‐ethylmaleimide‐sensitive factor Attachment Protein Receptors), which facilitate the fusion of insulin granules with the plasma membrane [1]. Among the SNARE proteins, SNAP25 (Synaptosomal‐associated protein 25) is pivotal in the exocytosis of insulin granules. It forms a core complex with other SNAREs, such as syntaxin 1A and VAMP2, enabling the precise docking and fusion of insulin granules [2]. By modulating the dynamics of granule release, SNAP25 plays an essential role in regulating insulin secretion.

One hallmark of type 2 diabetes (T2D) is β‐cell secretory dysfunction, but the underlying mechanisms are not fully understood. Notably, SNAP25 expression is reduced in islets from both T2D patients [3] and animals [4, 5, 6]. Consequently, the deficiency of SNAP25 impairs insulin secretion [7]. Conversely, overexpression of SNAP25 has been shown to improve insulin secretion in β‐cells exposed to chronic high glucose [7]. These findings suggest that restoring SNAP25 expression could benefit the treatment of T2D. Nevertheless, how the expression of SNAP25 is regulated remains elusive.

T2D is distinguished by hyperglycemia, which triggers oxidative stress in the β‐cells and is considered to be a detrimental factor contributing to β‐cell secretory dysfunction [8, 9]. Carbohydrate response element‐binding protein (ChREBP), a transcription factor sensitive to glucose levels, is expressed in pancreatic islets [10]. Elevated glucose activates ChREBP, which in turn regulates glucose‐induced gene expression [11]. Upon activation, ChREBP binds to the carbohydrate response element (ChoRE) on the target gene promoter, recruiting histone modifiers that lead to posttranslational modifications of histones and subsequently influence the expression of the target gene [12]. Additionally, the phosphorylation of Ser196 retains ChREBP in the cytoplasm and inhibits its transcription activity [13]. While ChREBP has been demonstrated to regulate insulin secretion [11], the precise mechanisms underlying these processes are still not entirely understood.

Analysis of DNA sequences revealed the presence of ChoRE in the *Snap25* promoter. This led to the hypothesis that ChREBP may regulate SNAP25 expression. In this study, we found that diabetic hyperglycemia suppresses SNAP25 expression, an effect associated with the activation of ChREBP, leading to increased ChREBP recruitment to the promoter of *Snap25*, and subsequently reducing the tri‐methylation of histone H3 at lysine 4 (H3K4me3) at the promoter.

## Materials and methods

### Experimental animals

Male Goto‐Kakizaki (GK) and Wistar rats (aged 8 weeks) were purchased from SLRC (Shanghai, China). The animal procedures comply with the approved Principles of Laboratory Animal Care by the Shenzhen University Animal Care Committee (Approval No.: IACUC‐202300082).

### Determination of blood glucose

Non‐fasting blood glucose levels in Wistar and GK rats were examined every week. Briefly, the blood samples were obtained from the tail vein of rats, then glucose levels were determined using a glucometer (Roche, Basel, Switzerland).

### Determination of plasma insulin

After fasting overnight, the rats were administered 1.5 g·kg^−1^ glucose via the jugular vein. Blood samples were collected at 0, 5, 10, and 30 min after glucose administration. Plasma insulin was determined using an Insulin (Rat) Ultrasensitive ELISA kit (80‐INSRTU‐E01; ALPCO, Salem, NH, USA).

### Islet isolation

The islets were isolated from rats as reported previously [14]. In brief, collagenase P (11249002001; Roche, Basel, Switzerland) was dissolved in Hank's balanced salts solution to achieve a concentration of 1.2 mg·mL^−1^. The rats were anesthetized and euthanized with excess chloral hydrate, and collagenase P was transfused into the pancreas via the common bile duct. After digestion, the islets were handpicked under a stereomicroscope and collected for western blot analysis.

### Cell lines

INS‐1 832/13 cells (passages 50–80) were cultured as described [14]. SNAP25 knockdown INS‐1 832/13 cells were generated as described previously [7]. pLV‐EF1α‐blast‐SNAP25 and pMSCV‐puro‐ChREBP plasmids were generated by inserting rat *Snap25* coding sequence or mouse ChREBP (*Mlxipl*) coding sequence into pLV‐EF1α‐blast vector or pMSCV‐puro plasmid, respectively. Subsequently, INS‐1 832/13 cells were transduced with the pLV‐EF1α‐blast‐SNAP25 or pMSCV‐puro‐ChREBP plasmid for SNAP25 or ChREBP overexpression, respectively, followed by selection with 3 μg·mL^−1^ blasticidin or puromycin for 1 week. For ChREBP knockdown, INS‐1 832/13 cells were lentivirally transduced with either scramble or shRNA targeting ChREBP (Cat. RMM3981‐201916728) from GE Dharmacon (Lafayette, CO, USA).

### Construction of luciferase plasmid and promoter reporter assay

The promoter fragment of rat *Snap25* (−948 to −554 bp) was amplified by PCR using the primers: forward 5′‐GGGGTACCCTGATGAATACCCTGGAA‐3′, reverse 5′‐CCCAAGCTTGCTACCTTGGACCCTAAT‐3′. Subsequently, the PCR product was cloned into a pGL3‐basic plasmid (Promega, Madison, WI, USA). For the determination of *Snap25* promoter activity, 293T cells were cotransfected with pGL3‐Snap25 and Renilla luciferase plasmids, followed by treatment with 11.1 mm glucose or 30 mm glucose medium for 48 h. The promoter activity was measured using the Dual‐Luciferase Reporter Assay kit (E1910; Promega, Madison, WI, USA).

### Western blot analysis

Approximately 50 μg protein purified from rat islets or INS‐1 832/13 cells was used in western blot [14]. These antibodies were used:anti‐SNAP25 (ab105105, 1 : 3000; Abcam, Cambridge, MA, USA), anti‐phospho‐ChREBP (1 : 5000; Proteintech, Wuhan, China), anti‐ChREBP (NBP2‐44307, 1 : 2000; NOVUS, Littleton, CO, USA), anti‐GAPDH (#5174, 1 : 3000; Cell Signaling, Danvers, MA, USA), anti‐histone H3 (ab1791, 1 : 3000; Abcam, Cambridge, MA, USA), and anti‐β‐actin (A5441, 1 : 10 000; Sigma‐Aldrich, St. Louis, MO, USA). The densities of the immunoblot bands were determined using gel‐pro analyzer 4.0 software (Media Cybernetics, Bethesda, MD, USA). The β‐actin or GAPDH or histone H3 was used as an internal control.

### Real‐time PCR analysis

RNA was extracted from INS‐1 832/13 cells using Trizol reagent (15596018CN; Invitrogen, Carlsbad, CA, USA) according to the instructions. One microgram of total RNA was used to prepare cDNA using a PrimeScript™ RT reagent Kit (RR047A; TaKaRa, Tokyo, Japan). Real‐time PCR analysis was conducted using ABI quantstudio 5 (Thermo Fisher Scientific, Waltham, MA, USA). The following primers were used: *Snap25*, forward 5′‐ACGCATTGAGGAAGGGAT‐3′, reverse 5′‐TCTGGCGATTCTGGGTGT‐3′; *Actin* (beta), forward 5′‐GTAAAGACCTCTATGCCAACA‐3′, reverse 5′‐GGACTCATCGTACTCCTGCT‐3′. The mRNA expression of SNAP25 was normalized to the internal control of β‐actin.

### Chromatin immunoprecipitation assay

Chromatin immunoprecipitation (ChIP) assay was performed using a ChIP assay kit (17‐295; Sigma‐Aldrich, St. Louis, MO, USA) as described [15]. Briefly, INS‐1 832/13 cells were cultured in 5.5 mm glucose medium (G5.5) or 30 mm glucose medium (G30) for 3 days, followed by fixing with 4% formaldehyde, then the cells were used to prepare DNA fragments. The fragments were incubated with 3 μg antigen‐specific antibody overnight at 4 °C, followed by an additional incubation with 65 μL protein A agarose beads for 3 h. DNA fragments were quantified by qPCR using the primers: forward 5′‐AGCACAACCCTCTGTTAA‐3′, reverse 5′‐CCAGCCTATTCTATTCTT‐3′. The percentage of the promoter bound by ChREBP or H3K4me3 in the total DNA (Input) was plotted and expressed as % Input.

### Insulin measurements

Insulin secretion was performed as described previously [14]. In brief, INS‐1 832/13 cells were preincubated in 1 mL KRB buffer for 1 h at 37 °C, followed by stimulation with 1 mL KRB buffer containing 2.8 mm glucose or 16.8 mm glucose for 30 min. The supernatants were collected for the determination of secreted insulin, and the attached cells were harvested for examination of cellular protein content. Insulin levels were determined by the rat insulin ELISA kit (80‐INSRTU‐E01; ALPCO, Salem, NH, USA). Insulin levels (ng·mL^−1^) were normalized against cellular protein content in mg.

### Statistical analyses

Data are presented as mean ± SD for the indicated number of experiments (*n*). The statistical significance for comparing two groups was determined using the independent *t*‐test, while for comparing multiple groups, the one‐way ANOVA with the least significant difference (LSD) *post hoc* test was utilized. A *P*‐value of less than 0.05 was deemed to be statistically significant.

## Results

### SNAP25 regulates insulin secretion and its expression is decreased in islets from GK rats

**Fig. 1:**
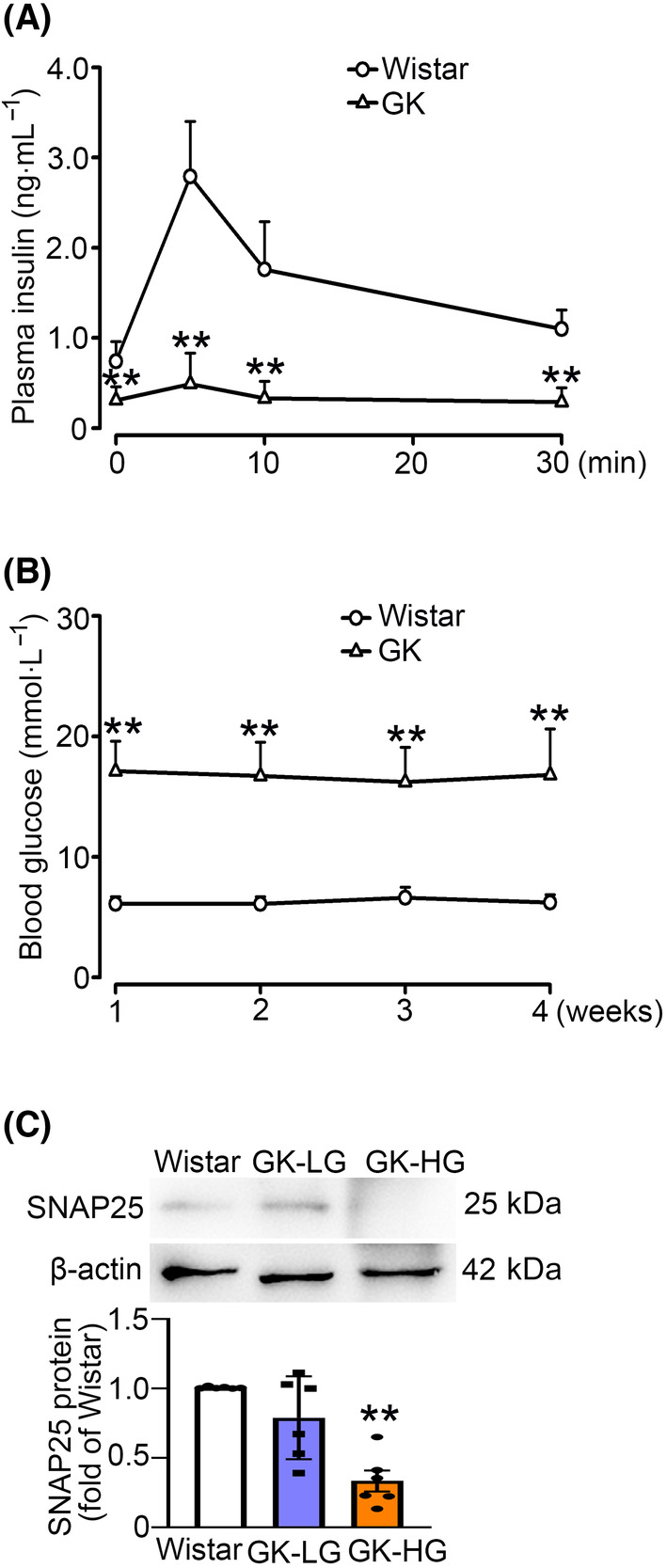
SNAP25 expression is decreased in islets from GK rats. (A) Plasma insulin in Wistar and GK rats. Data are mean ± SD of six rats per group. **P < 0.01. (B) Plasma glucose in Wistar and GK rats. Data represent mean ± SD of six rats per group. **P < 0.01. (C) SNAP25 protein expression in islets from Wistar and GK rats in prediabetic (blood glucose < 8 mmol·L^−1^, GK‐LG) and diabetic (blood glucose > 15 mmol·L^−1^, GK‐HG) states. β‐actin was used as the internal and loading control. Data are mean ± SD of six rats per group. **P < 0.01 vs. Wistar rats. Unpaired *t*‐test was used for statistical analysis of (A, B). One‐way ANOVA with LSD *post hoc* test was utilized for statistical analysis of (C).

Consistent with the previous study [15], GK rats displayed impaired insulin secretion (Fig. 1A) and hyperglycemia (Fig. 1B). Analysis of SNAP25 expression in islets from Wistar and GK rats showed a blood glucose‐dependent reduction in SNAP25 expression in GK rat islets (Fig. 1C).

**Fig. 2:**
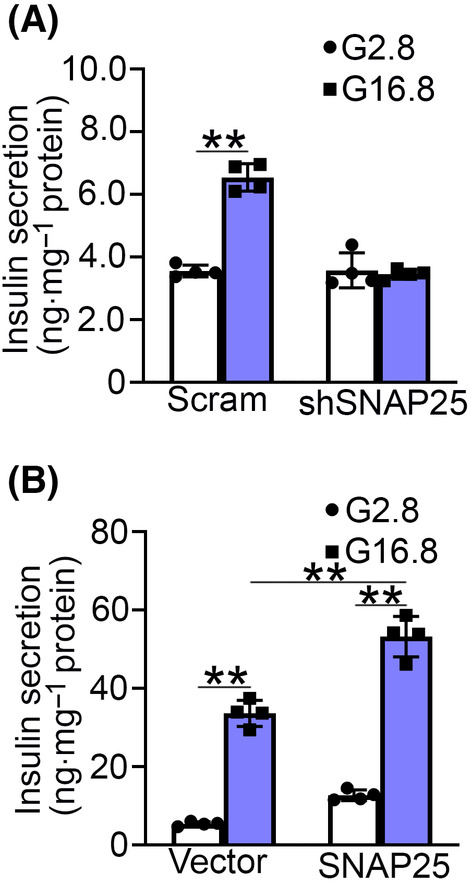
SNAP25 regulates insulin secretion in INS‐1 832/13 cells. (A) The scramble and shSNAP25 INS‐1 832/13 cells were stimulated with KRB buffer containing 2.8 mm (G2.8) or 16.8 mm (G16.8) glucose for 30 min. Insulin release was determined by ELISA assay. Data are mean ± SD from four independent experiments. **P < 0.01. (B) The vector and SNAP25 overexpressing INS‐1 832/13 cells were treated with G2.8 or G16.8 for 30 min. Insulin level was determined by ELISA. Data are mean ± SD from four independent experiments. **P < 0.01. One‐way ANOVA with LSD *post hoc* test was used for statistical analysis of (A, B).

To investigate the impact of reduced SNAP25 on the glucose‐stimulated insulin secretion (GSIS) of β‐cells, the scramble and SNAP25 knockdown (shSNAP25) INS‐1 832/13 cells were constructed, and insulin secretion was determined in these cells. Consistent with the previous study [7], shSNAP25 attenuated insulin secretion (Fig. 2A). In contrast, overexpression of SNAP25 enhanced GSIS in INS‐1 832/13 cells (Fig. 2B). These data corroborated the key role of SNAP25 in insulin secretion of β‐cells.

### ChREBP is activated in diabetic situations and inhibits insulin secretion and synthesis in INS‐1 832/13 cells

**Fig. 3:**
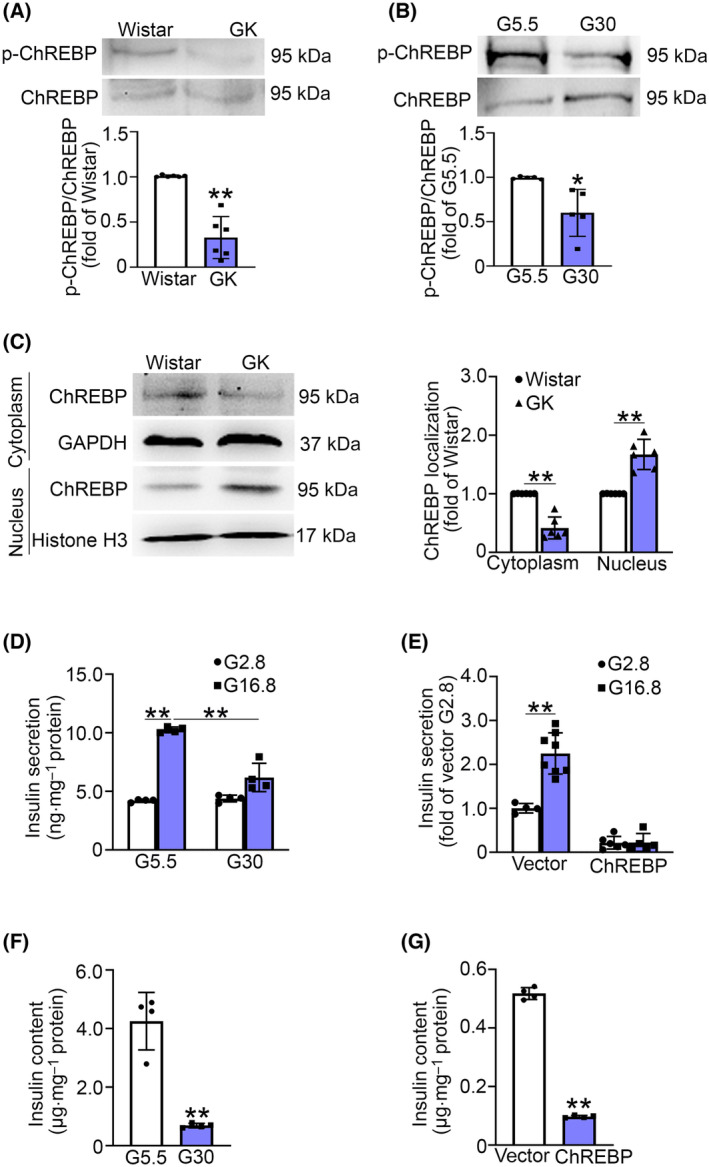
ChREBP is activated in diabetic β‐cells and suppresses insulin secretion and synthesis in INS‐1 832/13 cells. (A) The level of phosphorylation (Ser196) of ChREBP and total ChREBP in islets isolated from Wistar and GK rats. Intensities were quantified and normalized against the level of ChREBP and expressed as the percentage of protein abundance of Wistar islets. Data are mean ± SD of six rats per group. **P < 0.01. (B) The phosphorylation level of ChREBP in INS‐1 832/13 cells cultured in 5.5 mm (G5.5) or at 30 mm (G30) glucose medium for 3 days. Data are mean ± SD from five independent experiments. *P < 0.05. (C) The distribution of ChREBP in cytoplasm and nucleus in the pancreata of Wistar and GK rats. GAPDH and histone H3 were used as the cytosolic and nuclear loading control, respectively. Data are mean ± SD of six rats per group. **P < 0.01. (D) INS‐1 832/13 cells were cultured in G5.5 or G30 medium for 3 days, followed by stimulation with G2.8 or G16.8 for 30 min. Insulin level was determined by an ultrasensitive insulin ELISA kit. Values are normalized to total cellular protein at the respective glucose concentration. Data are mean ± SD from four independent experiments. **P < 0.01. (E) Similar to (D), but insulin secretion assay was performed in vector and ChREBP overexpressing INS‐1 832/13 cells. Data are mean ± SD from four (vector‐G2.8), eight (vector‐G16.8), six (ChREBP‐G2.8), or five (ChREBP‐G16.8) independent experiments. **P < 0.01. (F) INS‐1 832/13 cells were cultured in the condition same as (D). Insulin content was examined by ELISA assay and was normalized against total cellular protein content in mg. Data are mean ± SD from four independent experiments. **P < 0.01. (G) Insulin content was examined in the vector and ChREBP overexpressing INS‐1 832/13 cells. Data represent mean ± SD from four independent experiments. **P < 0.01. Unpaired *t*‐test was used for statistical analysis of (A–C, F, G). One‐way ANOVA with LSD *post hoc* test was employed for statistical analysis of (D, E).

ChREBP, a transcriptional factor that senses glucose levels, has been demonstrated to mediate the harmful effects of high glucose [11]. Subsequently, we investigated the activity of ChREBP in islets from Wistar and GK rats by examining its phosphorylation. Our analysis revealed that ChREBP phosphorylation in GK islets was approximately 67% lower than that in the Wistar controls (Fig. 3A). The decreased ChREBP phosphorylation was also observed in INS‐1 832/13 cells exposed to 30 mm glucose for 3 days (Fig. 3B), indicating the activation of ChREBP in diabetic conditions. We then investigated the subcellular distribution of ChREBP and found that ChREBP predominantly localized in the nucleus in the pancreata of GK rats compared to Wistar rats (Fig. 3C).

To assess the impact of activated ChREBP on insulin secretion, we cultured INS‐1 832/13 cells in a medium containing either 5.5 mm glucose (G5.5) or 30 mm glucose (G30) for 3 days, followed by the determination of insulin release upon stimulation with 2.8 mm glucose (G2.8) or 16.8 mm glucose (G16.8) for 30 min. The results showed that G16.8 stimulation produced a ~ 2.4‐fold increase in insulin secretion in INS‐1 832/13 cells cultured in the G5.5 medium. However, prolonged activation of ChREBP by chronic G30 culture led to diminished insulin secretion (Fig. 3D). Similar observations were also made in ChREBP overexpressing INS‐1 832/13 cells (Fig. 3E), which are consistent with the previous study [11]. We further investigated the effect of ChREBP on insulin content in INS‐1 832/13 cells. The results showed that both the activation of ChREBP by high glucose (Fig. 3F) and the overexpression of ChREBP (Fig. 3G) led to approximately 80% decrease in insulin content. These findings suggest that ChREBP plays a critical role in the process of insulin synthesis and secretion, and its overactivation may be a crucial contributing factor to β‐cell secretory dysfunction in T2D.

### ChREBP mediates high glucose‐inhibited SNAP25 expression in INS‐1 832/13 cells

**Fig. 4:**
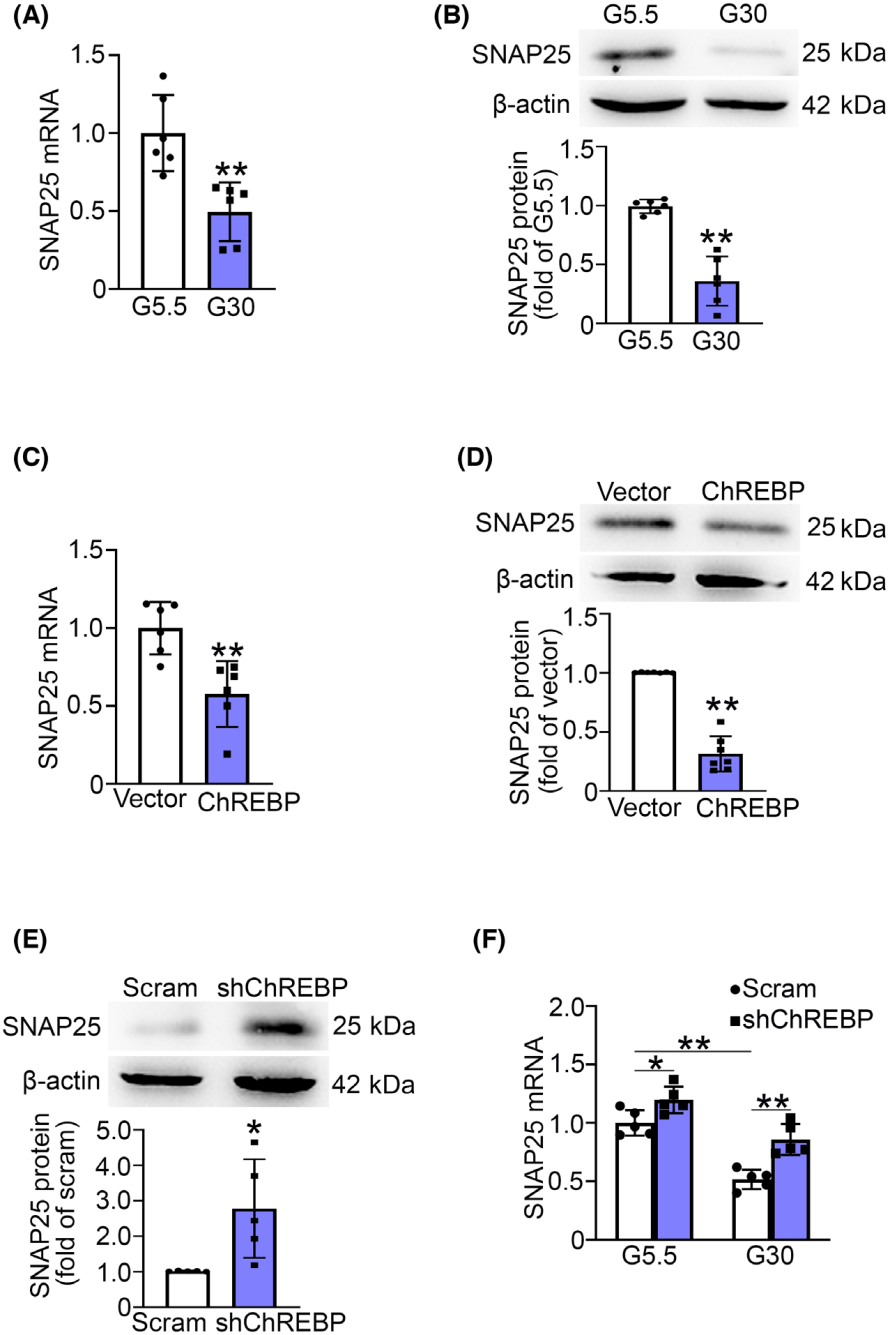
ChREBP negatively regulates SNAP25 expression in INS‐1 832/13 cells. (A, B) INS‐1 832/13 cells were cultured in G5.5 or G30 medium for 3 days. The mRNA (A) and protein (B) of SNAP25 were determined using qPCR and western blot assays, respectively. β‐actin was used as the internal control. Data are mean ± SD from six independent experiments. **P < 0.01. (C, D) The levels of mRNA (C) and protein (D) of SNAP25 were determined in the vector and ChREBP overexpressing INS‐1 832/13 cells. β‐actin was used as the internal and loading control. Data are mean ± SD of six (C) and seven (D) independent experiments. **P < 0.01. (E) SNAP25 protein was determined in the scramble and shChREBP INS‐1 832/13 cells. β‐actin was used as the internal and loading control. Data are mean ± SD from five independent experiments. *P < 0.05. (F) The scramble and shChREBP INS‐1 832/13 cells were cultured in 5.5 or 30 mm glucose medium for 3 days. Then SNAP25 mRNA was examined by qPCR. Data are mean ± SD from five independent experiments. *P < 0.05; **P < 0.01. The statistical analysis for (A–E) utilized an unpaired *t*‐test, while for (F) it involved one‐way ANOVA with LSD *post hoc* test.

To investigate the correlation between reduced SNAP25 expression in diabetic conditions and the hyperglycemia‐induced overactivation of ChREBP, INS‐1 832/13 cells were cultured in G5.5 or G30 medium for 3 days, followed by an assessment of the mRNA and protein levels of SNAP25. The results demonstrated that the activation of ChREBP due to high glucose led to approximately 50% and 60% lower levels of SNAP25 mRNA and protein, respectively, compared with the control cells (Fig. 4A,B). These findings supported the idea that hyperglycemia inhibits SNAP25 expression [16].

Subsequently, we analyzed the mRNA and protein expression of SNAP25 in vector and ChREBP overexpressing INS‐1 832/13 cells. The results showed that overexpression of ChREBP produced approximately 40% and 70% decrease in SNAP25 mRNA and protein levels, respectively, compared with the control cells (Fig. 4C,D). On the contrary, the knockdown of ChREBP (shChREBP) by shRNA resulted in a ~ 2.8‐fold increase of SNAP25 protein expression (Fig. 4E). These results suggested a negative regulatory role of ChREBP on SNAP25 expression.

Further investigation aimed to determine whether ChREBP mediates the inhibitory effect of high glucose on SNAP25 expression. The scramble and shChREBP INS‐1 832/13 cells were cultured in G5.5 or G30 medium for 3 days, followed by the analysis of mRNA expression of SNAP25. This revealed that shChREBP made a modest but significant increase in SNAP25 mRNA levels in low glucose conditions. Importantly, it was observed that shChREBP partially reversed the high glucose‐induced decrease in SNAP25 mRNA expression (Fig. 4F), indicating the crucial involvement of ChREBP in the reduction of SNAP25 expression under high glucose conditions.

### High glucose promotes ChREBP recruitment and reduces H3K4me3 at the *Snap25* promoter

**Fig. 5:**
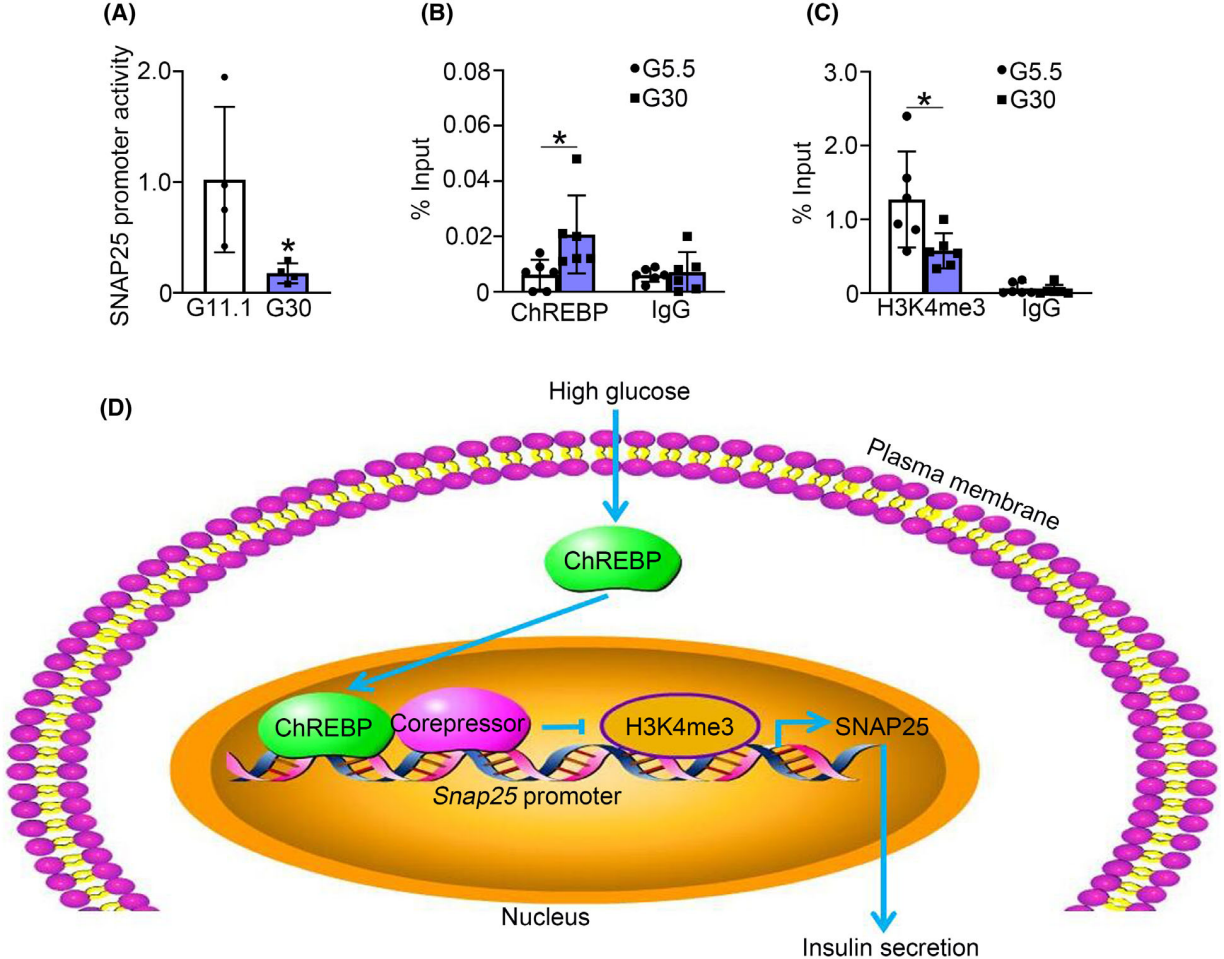
High glucose promotes ChREBP recruitment and decreases H3K4me3 at the *Snap25* promoter. (A) 293T cells were transfected with a luciferase reporter driven by *Snap25* promoter, followed by culture in G11.1 or G30 medium for 48 h before luciferase activity analysis for the reporter expression. Data are mean ± SD from four independent experiments. *P < 0.05. (B) ChIP assays were performed using anti‐ChREBP antibodies in INS‐1 832/13 cells treated with G5.5 or G30 for 72 h. IgG served as a negative control. Data are mean ± SD from six independent experiments. *P < 0.05. (C) ChIP assays were performed to determine the level of H3K4me3 at the promoter of *Snap25* in INS‐1 832/13 cells cultured in G5.5 or G30 for 72 h. IgG served as a negative control. Data are mean ± SD from six independent experiments. *P < 0.05. Unpaired *t*‐test was used for statistical analysis of (A). One‐way ANOVA with LSD *post hoc* test was utilized for statistical analysis of (B, C). (D) Proposed model of decreased SNAP25 expression in diabetic β‐cells. See text for details.

To clarify the mechanism of high glucose‐inhibited SNAP25 expression, we created a luciferase reporter driven by rat *Snap25* promoter and transfected it into 293T cells, followed by treatment with G30 and examination of the luciferase activity. The results showed that G30 treatment suppressed the promoter activity of the *Snap25* gene (Fig. 5A). Furthermore, ChIP assay revealed that high glucose promoted ChREBP binding to the promoter of *Snap25* (Fig. 5B). In addition, G30 treatment reduced the level of H3K4me3, a marker for stimulating gene expression, at the *Snap25* promoter (Fig. 5C).

## Discussion

This study demonstrates, for the first time, that ChREBP is responsible for the decrease in SNAP25 expression induced by high glucose, involving the reduction in H3K4me3 at the *Snap25* promoter. The reduced SNAP25 expression may be a crucial factor contributing to β‐cell secretory dysfunction and may stem from ChREBP overactivation in T2D.

SNAP25 plays an essential role in the process of insulin secretion of β‐cells [17], and its expression is reduced in islets during diabetic situations [3, 4, 5, 6], our data corroborate this phenomenon. Hence, the decreased SNAP25 expression may be a critical factor of β‐cell secretory dysfunction in T2D. It is noteworthy that hyperglycemia inhibits SNAP25 expression in β‐cells [16] (Fig. 4A,B). Given that ChREBP is a mediator of high glucose and plays a pivotal role in β‐cell glucotoxicity [11], we assessed the role of ChREBP in high glucose‐decreased SNAP25 expression in β‐cells. We demonstrated that SNAP25 is one of the downstream target genes negatively regulated by ChREBP. This opinion is supported by the evidence that high glucose‐activated or overexpressed ChREBP suppressed SNAP25 expression, while ChREBP knockdown increased it (Fig. 4A–E). Moreover, shChREBP partially reversed the decrease in SNAP25 expression induced by high glucose (Fig. 4F), indicating the crucial role of ChREBP in the process of high glucose inhibiting SNAP25 expression. Furthermore, our findings also revealed that high glucose‐induced ChREBP activation promoted ChREBP occupancy (Fig. 5B) and attenuated H3K4me3 at the *Snap25* promoter (Fig. 5C). This would lead to decreased *Snap25* gene expression, given that H3K4me3 facilitates enhanced transcription at the target DNA locus [18]. However, the precise molecular mechanism through which ChREBP mediates SNAP25 expression remains to be investigated.

It is reported that the activity of ChREBP is controlled by phosphorylation [19]. We confirmed that ChREBP is activated in diabetic islets (Fig. 3A). Consistent with previous studies [11], enhanced ChREBP activity suppressed insulin secretion, potentially stemming from decreased insulin content (Fig. 3F,G) and SNAP25 expression (Fig. 4A–D). The reduced insulin content is likely a result of the negative regulation of ChREBP on gene expression of *Pdx‐1*, *Mafa*, *Ins1*, and *Ins2* [20]. The reduction in SNAP25 expression in T2D may be attributed to the enhanced ChREBP signaling. Additionally, our recent study has revealed that SNAP25 expression is also regulated by GLP‐1 signaling [7]. Given the plasma GLP‐1 level and its receptor expression are decreased in T2D [21, 22], it is plausible that the combination of heightened glucose‐induced ChREBP activity and compromised GLP‐1 signaling contributes to the decreased expression of SNAP25 in diabetic β‐cells. Further investigation is needed to determine whether ChREBP is involved in GLP‐1 signaling‐mediated SNAP25 expression.

## Conclusion

Our data unravel the novel molecular mechanisms of reduced SNAP25 expression in diabetic β‐cells: ChREBP drives the downregulation of SNAP25 expression by regulating H3K4me3 at the promoter (Fig. 5D). These findings are important, as little was previously known about how SNAP25 expression is inhibited in diabetes. The decreased expression of SNAP25 contributes at least partially to the impaired insulin secretion of diabetic β‐cells. Further exploration of the ChREBP/SNAP25 pathway could help to identify new approaches for treating T2D.

## Conflict of interest

The authors declare no conflict of interest.

## Author contributions

AH, HL, ZY, and XK performed the experiments. XK designed the project, analyzed the data, and wrote the manuscript. All authors read and approved the final version of the manuscript.

## Data Availability

The data used in this study are available from the corresponding author upon request.
